# Beta-Blockers, Left and Right Ventricular Function, and *In-Vivo* Calcium Influx in Muscular Dystrophy Cardiomyopathy

**DOI:** 10.1371/journal.pone.0057260

**Published:** 2013-02-20

**Authors:** Alison Blain, Elizabeth Greally, Steve Laval, Andrew Blamire, Volker Straub, Guy A. MacGowan

**Affiliations:** 1 Institute of Genetic Medicine, Newcastle University, International Centre for Life, Central Parkway, Newcastle upon Tyne, United Kingdom; 2 Institute of Cellular Medicine and Newcastle Magnetic Resonance Centre, Campus for Ageing and Vitality, Newcastle University, Newcastle upon Tyne, United Kingdom; 3 Department of Cardiology, Freeman Hospital, Newcastle upon Tyne, United Kingdom; University of Buenos Aires, Cardiovascular Pathophysiology Institute, Argentina

## Abstract

Beta-blockers are used to treat acquired heart failure in adults, though their role in early muscular dystrophy cardiomyopathy is unclear. We treated 2 different dystrophic mouse models which have an associated cardiomyopathy (*mdx*: model for Duchenne Muscular Dystrophy, and *Sgcd*-/-: model for limb girdle muscular dystrophy type 2F) and wild type controls (C57 Bl10) with the beta blocker metoprolol or placebo for 8 weeks at an early stage in the development of the cardiomyopathy. Left and right ventricular function was assessed with cardiac magnetic resonance imaging (MRI) and *in-vivo* myocardial calcium influx with manganese enhanced MRI. In the *mdx* mice at baseline there was reduced stroke volume, cardiac index, and end-diastolic volume with preserved left ventricular ejection fraction. These abnormalities were no longer evident after treatment with beta-blockers. Right ventricular ejection fraction was reduced and right ventricular end-systolic volume increased in the *mdx* mice. With metoprolol there was an increase in right ventricular end-diastolic and end-systolic volumes. Left and right ventricular function was normal in the *Sgcd*-/- mice. Metroprolol had no significant effects on left and right ventricular function in these mice, though heart/body weight ratios increased after treatment. *In-vivo* myocardial calcium influx with MEMRI was significantly elevated in both models, though metoprolol had no significant effects on either. In conclusion, metoprolol treatment at an early stage in the development of cardiomyopathy has deleterious effects on right ventricular function in *mdx* mice and in both models no effect on increased *in-vivo* calcium influx. This suggests that clinical trials need to carefully monitor not just left ventricular function but also right ventricular function and other aspects of myocardial metabolism.

## Introduction

In Duchenne muscular dystrophy the absence of dystrophin causes subclinical or clinical dilated cardiomyopathy [1], [2]. Also, mutations in one of the genes for α-, β-, γ- or δ-sarcoglycan, a heterogeneous group of autosomal recessive limb girdle muscular dystrophies (LGMD2C-F), cause severe dilated cardiomyopathy at a young age in addition to muscular dystrophy, especially in those patients with LGMD2F caused by mutations in the δ-sarcoglycan gene [1]–[3].

Beta-blockers are established treatments for acquired heart failure [4] though the role in inherited cardiomyopathies is less clear. It has been suggested that for certain dystrophin mutations which predispose to cardiomyopathy, early treatment including beta-blockers is of benefit [5]. We have shown that in *mdx* mice, which lack the protein dystrophin as in Duchenne muscular dystrophy that there are benefits in terms of left ventricular pressure-volume derived data with early beta- blocker treatment, though detrimental effects in the delta sarcoglycan deficient mouse model (*Sgcd*-/-, model for limb girdle muscular dystrophy type 2F) [6]. This suggests that not all underlying genotypes may respond beneficially to beta-blockers.

However, left ventricular pressure and volume measurements are not the only physiological measurements that can assess the effects of a systemic treatment. Right ventricular function is frequently abnormal in cardiomyopathy. Both left and right ventricular function can be assessed non-invasively with cardiac magnetic resonance imaging (MRI). Increased intracellular calcium is an important feature of muscular dystrophy cardiomyopathy and plays a central role in its pathophysiology [7]. Manganese-enhanced magnetic resonance imaging (MEMRI) can assess *in-vivo* calcium influx using 2 properties of the manganese ion [8]. Manganese enters cardiomyocytes through calcium channels, and is also a T_1_ contrast agent with MRI. Thus, manganese influx results in a relative increase in contrast-enhancement on T_1_ weighted images.

The purpose of this study was to treat 2 different mouse models of muscular dystrophy cardiomyopathy with beta-blockers at an early stage in the development of the cardiomyopathy to determine if there are beneficial effects on left and right ventricular function and to determine whether beta-blockers reduced *in-vivo* myocardial calcium influx.

## Materials and Methods

### Animals

Male *mdx* (n = 15) from Jackson laboratories (Bar Harbor, ME, USA), *Sgcd*-/- mice (n = 11) and C57 Bl10 mice (Jackson Laboratories) (n = 6) were treated with metoprolol and compared to age matched controls (*mdx* (n = 21), *Sgcd*-/- (n = 20), C57 Bl10 (n = 19)). Mice were housed under controlled temperature (17–28°C) and light conditions (12:12h light: dark cycle). Animals had free access to food and water. The investigations conformed with the Guide for the Care and Use of Laboratory Animals published by the US National Institutes of Health [NIH Publication No.85–23, revised in 1985 and was performed under the terms of the Animals (Scientific Procedures) Act 1986, authorized by the Home Secretary, Home Office UK]. All experiments were performed at the animal care facility of Newcastle University, UK. The work was approved by the Ethical Review Committee of Newcastle University.

### Drug treatment

Metoprolol (2.5 mg/kg/day) (M5391, Sigma-Aldrich) was administered orally via the drinking water, which was refreshed every 48 hours. From our previous studies [9]–[11] we have been able to establish that mice (housed under the conditions in our animal unit) drink an average volume of 2.5 ml/day drinking water. Mice were weighed fortnightly and the concentration of metoprolol in the drinking water was adjusted accordingly. Treatment was initiated in the *mdx* mice at 16 weeks and at 8 weeks in the *Sgcd*-/- mice, timepoints which are early in the development of left ventricular dysfunction [12]. Treatment was continued for 8 weeks and cardiac function was assessed in the *mdx* mice at 24 weeks of age and 16 weeks in *Sgcd*-/- mice; ages at which cardiac function has previously been shown to be impaired in these mouse models [6], [10]. The C57 Bl10 mice were aged 12 weeks at the start of the treatment as an intermediate age between the *mdx* and *Sgcd*-/- mice.

### MRI

Following induction of anaesthesia with 5% isofluorane in an anaesthetic chamber, mice were cannulated via the tail vein and laid prone on a cradle that allowed monitoring of body temperature, respiratory rate and heart rate heart rate (Dazai Research Instruments, Toronto, Canada). Mice were placed in the magnet and anaesthesia was maintained using 1–1.8% isofluorane via a nose cone. Their body temperature was maintained using a warm air blower. Images were acquired on a 7 Tesla horizontal bore Varian microimaging system equipped with a 12-cm microimaging gradient insert (maximum gradient 40 gauss/cm), (Varian Inc., Palo Alto, CA, USA). Mice were scanned in a 39 mm diameter quadrature birdcage volume coil (Rapid Biomedical GmbH). Following power calibration and global shimming a series of four pilot transverse images were acquired over the heart. Single slice coronal and sagittal images were then obtained in order to view the apex and mitral valve planes. These images were used to plan for the true short axis plane. To measure ventricular function, up to twelve contiguous short axis slices were acquired to cover the entire left and right ventricles using a spoiled gradient-echo cine sequence (TR = 5 ms, TE = 1.42 ms, flip angle 15°, FOV 30×30 mm, data matrix 128×128, 1 mm slice thickness). Images were ECG triggered to the R wave with a cine delay of 15 ms and typically 30 phases were acquired distributed through the cardiac cycle. Images were zero-filled to a matrix size of 256×256. Scans were converted to matfiles using a matlab script (kindly provided by Johannes Riegler, UCL) and analysed using the freely available analysis software Segment v1.8 (http://segment.heiberg.se) to give LV and RV functional parameters. Segment automatically delineated the endocardial and pericardial borders at end systole and end diastole and manual adjustments were made if necessary. LV mass, RV and LV volumes, ejection fractions and cardiac output were calculated.

### Manganese enhanced MRI (MEMRI)

Manganese chloride (60 mM, Sigma-Aldrich 244589) was given by intravenous infusion through the tail vein canula at a flow-rate of 0.6 ml/hour. Flow time was adjusted according to weight to give a total dose of 190 nmol/g body weight (for example, for a 30 g mouse, this would result in a 9.5 minute infusion). Single slice gradient echo short axis images at the level of the papillary muscles (T_1_ weighted parameters: TR = 35 ms, TE = 3.5 ms, flip angle 60°, FOV 30 mm×30 mm, data matrix 128×128, 1 mm slice thickness, 6 averages) were taken. Prior to manganese infusion four baseline images were acquired in order to average any variations due to changes in TR as a result of fluctuations in heart rate. Infusion was begun and images were taken at 2.5 mins, 5 mins and then at 5 minute intervals thereafter, for 30 minutes. A relative increase in T_1_ weighted contrast indicates increased manganese uptake. The time course stack of images were analysed in ImageJ (http://rsb.info.nih.gov/ij). An area of interest was drawn to fit inside the myocardium and average signal intensity was measured. If necessary, minor adjustments of this drawn region were made for subsequent images in the stack and the increase in myocardial contrast enhancement was expressed as a percentage increase from the average of the four baseline images (which showed little or no variation). MEMRI data is expressed as an enhancement ratio over baseline.

### Statistics

Where appropriate, data was normalized to body weight. All data are reported as mean ± [standard error]. Differences between animal and treatment groups were compared by univariate general linear model ANOVA with Bonferroni-Sidak posthoc testing, and statistically significant findings of *P<0.05* are reported.

## Results

### Ventricular hypertrophy in untreated *mdx* mice and metoprolol-treated *Sgcd*-/- mice

**Figure 1:**
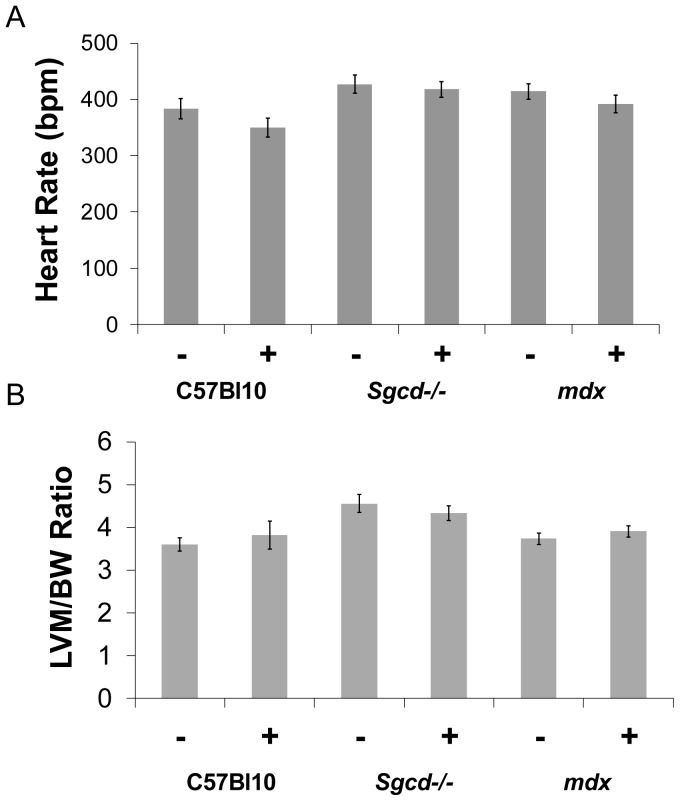
Heart rates and left ventricular mass to body weight ratio. (A) Mean anesthetized heart rates in all groups; (BPM = beats/min, – sign indicates without metoprolol and + indicates with). (B) Left ventricular mass to body weight ratio as determined by MR imaging is not affected by metoprolol treatment (LVM = left ventricular mass, BW = body weight, – sign indicates without metoprolol and + indicates with).

Metoprolol treatment was well tolerated and there were no early deaths in the treated mice. *Sgcd*-/- mice had lower body weights compared to C57 Bl10 controls (table 1). Compared to untreated C57 Bl10 mice, *mdx* mice had evidence of ventricular hypertrophy (post mortem combined weight of right and left ventricles) with elevated heart to body weight ratios with and without metroprolol, and the *Sgcd*-/- mice had an increased heart to body weight ratio after metroprolol only (table 1). Mean anesthetized heart rates for all groups are shown in Figure 1a.

**Table 1:** Body and heart weights of metoprolol treated mice.

	C57 Bl10	C57Bl10 metoprolol	*Sgcd*-/-	*Sgcd*-/- metoprolol	*Mdx*	*mdx* metoprolol
Body weight (g)	32.6 (0.72)	31.7 (0.76)	29.3 (0.63) *	29.1 (0.53) *	35.6 (0.55)	33.2 (1.08) ^$$^
Heart weight (mg)	125.8 (2.73)	132.0 (6.94)	131.6 (3.95)	143.2 (4.70)	156.4 (4.30)*** ^$$^	151.8 (6.53) *
Heart weight to body weight ratio	3.9 (0.12)	4.2 (0.26)	4.4 (0.13)	4.9 (0.09) ***	4.4 (0.09)*	4.5 (0.14) *

Mean+SEM. * different from C57 B10 control, $ different from *Sgcd*-/- control. Number of symbols denotes level of significance e.g. *p<0.05, **p<0.01, ***p<0.001

In *mdx* mice metoprolol has beneficial effects on left ventricular size and function though detrimental effects on right ventricular size, whilst in *Sgcd*-/- mice metroprolol increases left ventricular size

#### C57 Bl10

Beta-blockers had no significant effects on left or right ventricular function or left ventricular mass.

#### Mdx

**Figure 2:**
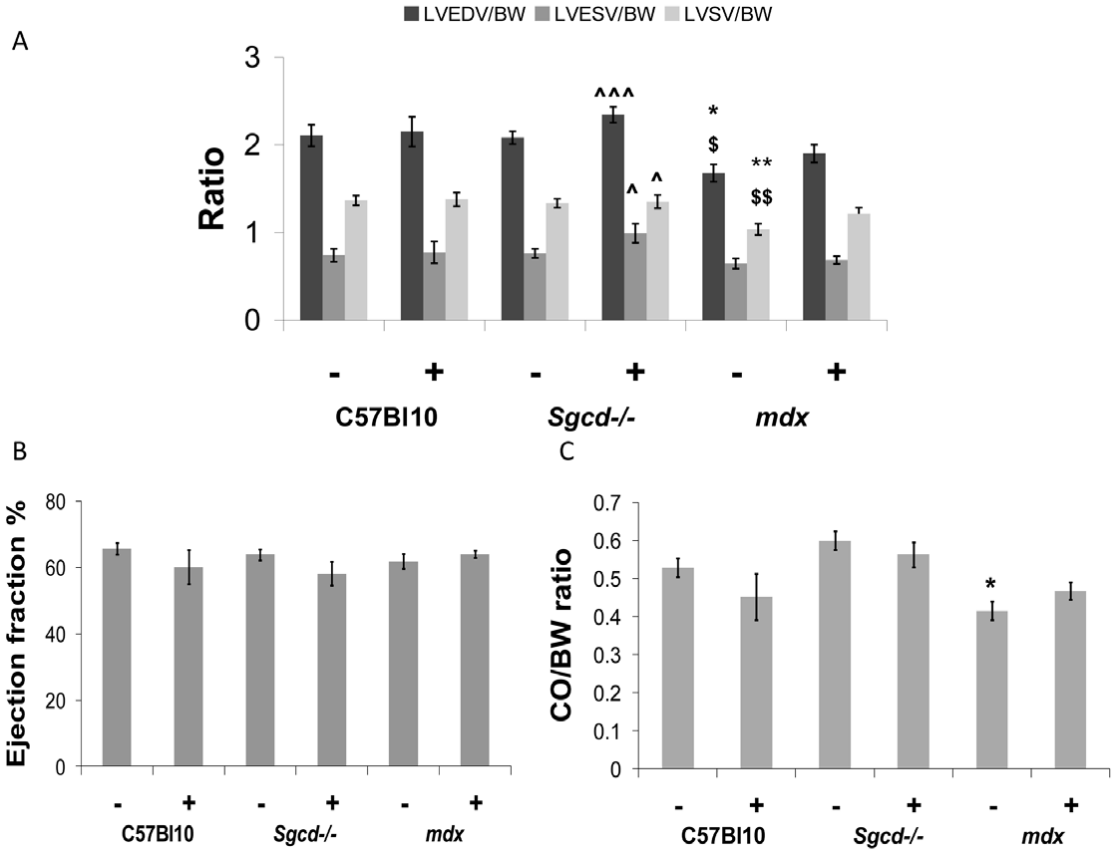
Effect of metoprolol treatment on left ventricular function. (A) Left ventricular volume to body weight (BW) ratios; (B) Ejection fraction; (C) Cardiac output (CO) to body weight ratio. Reductions in left ventricular end-diastolic and (LV EDV) and left ventricular stroke volume index (LV SV) in untreated *mdx* mice were no longer evident after metoprolol treatment. In untreated *Sgcd-/-* mice there was normal left ventricular function, and after treatment with metropolol there were increases in end-diastolic, end-systolic (LV ESV) and stroke volume indexes relative to untreated *mdx* mice. (*different from C57 Bl10 control, ^$^different from *Sgcd-/-* control, ?different from *mdx* control; number of symbols denotes level of significance e.g. *p<0.05, **p<0.01, ***p<0.001); (– sign indicates without metoprolol and + indicates with).

**Figure 3:**
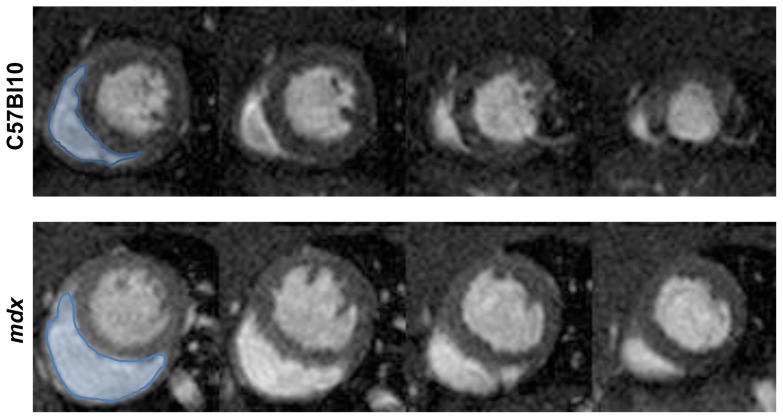
*Mdx* mice have RV dilatation. Representative 2 chamber short axis cine slices at the level of the papillary muscles (basal to apical slices from left to right) at end diastole in *mdx* and C57 Bl10 mice. The right ventricle is highlighted in blue in the first slices.

**Figure 4:**
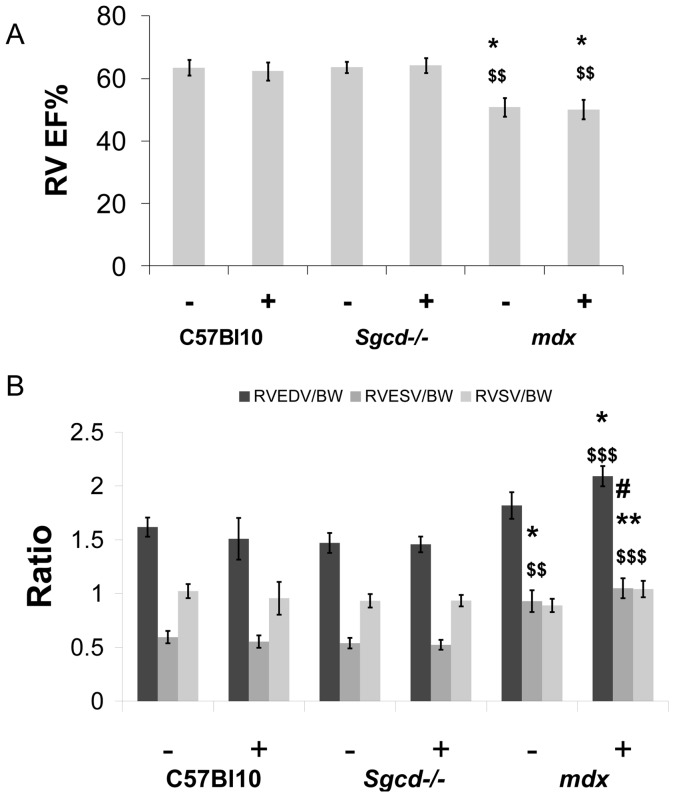
Deterioration in right ventricular function with metoprolol in mdx mice. Right ventricular ejection fraction (RV EF, panel A), and right ventricular volume data (with abbreviations as for LV in figure 2, panel B). (*Different from C57 Bl10 control, ^$^different from *Sgcd-/-* control, ^#^different from metoprolol-treated C57 Bl10, number of symbols denotes level of significance e.g. *p<0.05, **p<0.01, ***p<0.001); (BW = body weight, – sign indicates without metoprolol and + indicates with).

The cardiomyopathy in the untreated *mdx* mice was characterized by a smaller left ventricular cavity in diastole, reduced stroke volume and cardiac output although left ventricular mass (determined by MR imaging) and left ventricular ejection fraction were not significantly different from controls (Figures 1b and 2). Metoprolol had beneficial effects on left ventricular function in *mdx*; stroke volume index (Figure 2a), end-diastolic left ventricular volume index (Figure 2a) and cardiac output index (Figure 2c) were all normalized towards C57 Bl10 values. Right ventricular dysfunction was not significantly improved by treatment however. *Mdx* mice have marked right ventricular dysfunction with reduced right ventricular ejection fraction and increased right ventricular end-systolic volume at baseline (Figure 3, and 4a and b). Metoprolol did not normalize these parameters and indeed there was a further increase in right ventricular end-diastolic volume such that this parameter was now significantly higher than C57 Bl10 and *Sgcd*-/- controls, and right ventricular end-systolic volume was significantly higher than metoprolol-treated C57 Bl10 mice (Figure 4b).

#### *Sgcd*-/-

The untreated *Sgcd*-/- mice had normal left and right ventricular systolic function and left ventricular mass when compared with C57 Bl10 controls (Figures 1 and 2). There was some evidence of change in left ventricular function in the *Sgcd*-/- mice with metoprolol; in addition to the increased heart weight to body weight ratio (Table 1), the left ventricular end-diastolic, end-systolic and stroke volume indexes (Figure 2a) were significantly increased compared to untreated *mdx* mice, though not compared to C57 Bl10 mice. There were no significant changes in right ventricular function with metoprolol (Figure 4).

### Metoprolol treatment does not normalize increased *in-vivo* calcium influx in *mdx* or *Sgcd*-/- mice as measured by MEMRI

**Figure 5:**
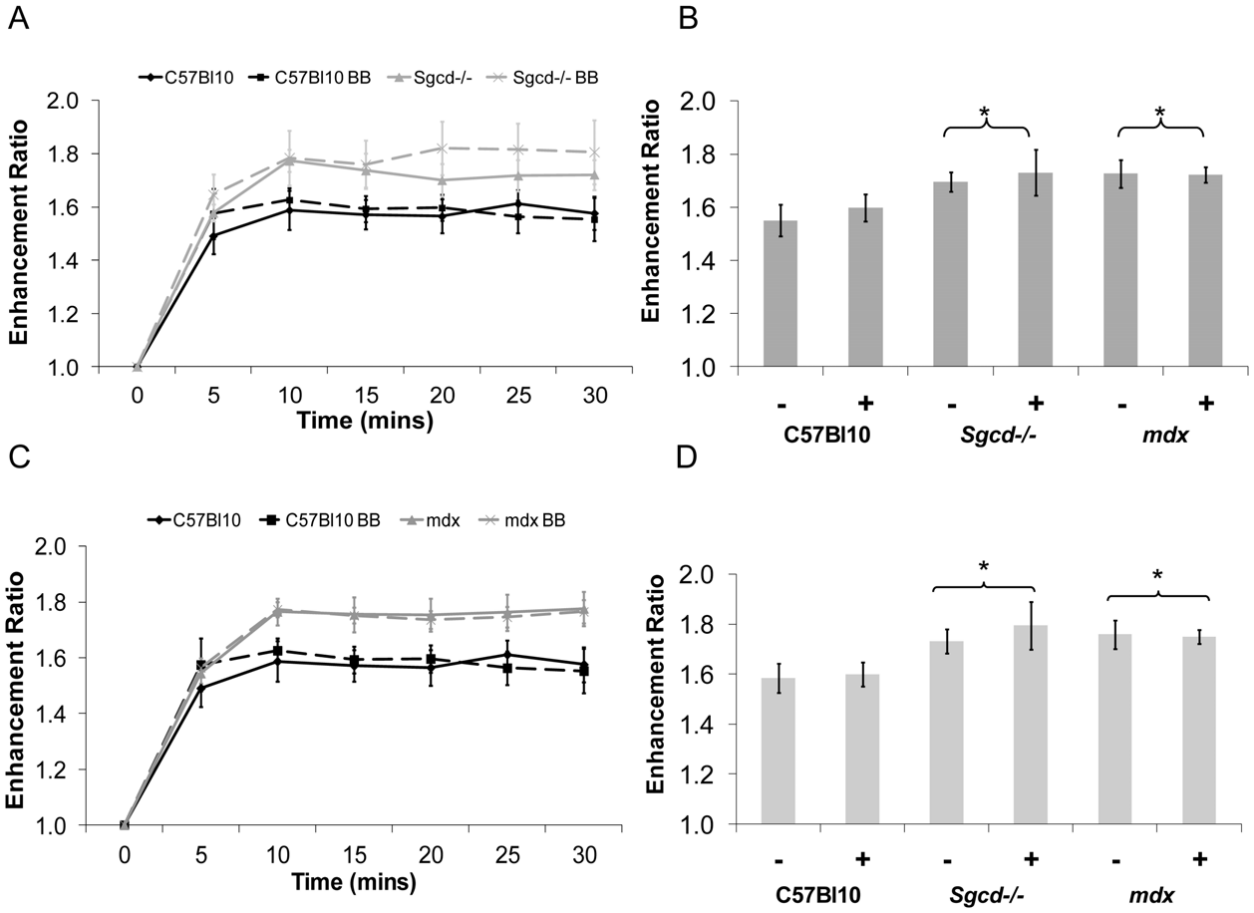
Metoprolol does not significantly alter increased manganese uptake in *Sgcd*-/- and *mdx* mice. There is a significant strain effect (p<0.05, ANOVA) on (B) early (5-15 mins) and (D) steady state (10-25 mins) manganese uptake enhancement ratio, such that Sgcd-/- (A) and *mdx* (C) mice have a significantly higher manganese uptake than C57 Bl10 mice. Beta-Blocker treatment has no significant effect on manganese uptake. MEMRI data is expressed as an enhancement ratio over baseline levels. (*p<0.05 vs C57 Bl10, – sign indicates without metoprolol and + indicates with).

There were no significant effects of beta-blocker treatment on the C57 Bl10 mice on manganese influx. Manganese influx was increased in both *mdx* and *Sgcd*-/- mice compared to C57 Bl10 mice at early (5 – 15 minutes) and steady state (10 – 25 minutes) stages following the start of the infusion (p<0.05, ANOVA) (Figure 5 a-d) as previously demonstrated [13], indicating *in-vivo* increased calcium influx occurring at an early stage in the development of the cardiomyopathy. This was not significantly affected in either group by treatment with metoprolol (p = ns, ANOVA).

## Discussion

In two mouse models of muscular dystrophy cardiomyopathy treated with the beta-blocker metoprolol at an early stage in the development of the cardiomyopathy, we show that there are differences in left and right ventricular function and the responses to treatment. In the *mdx* mice without treatment there is ventricular hypertrophy, reduced left ventricular stroke volume with a small left ventricular cavity size, normal left ventricular ejection fraction but reduced right ventricular ejection fraction. Consistent with our previous invasive catheter based studies there are benefits of metroprolol on left ventricular function in the *mdx* mice, but here we also demonstrate deterioration in right ventricular function. The *Sgcd*-/- mice develop ventricular hypertrophy with metoprolol (compared to untreated C57 Bl10 mice), but have normal left and right ventricular function without treatment with no significant effects of treatment on left or right ventricular function relative to C57 Bl10 mice. Despite these heterogeneous patterns of ventricular function in the 2 models, both exhibit increased intracellular calcium influx that is unaffected by metoprolol. Thus, overall responses to metoprolol in both models are largely either absent or potentially detrimental.

### Right ventricular function

In clinical heart failure, right ventricular dysfunction is an important predictor of outcomes [14]. In muscular dystrophy cardiomyopathy, right ventricular dysfunction may be an important early feature. In Becker’s muscular dystrophy cardiac involvement appears to develop first in the right ventricle when seen in younger patients, and left ventricular involvement develops at a later age [15], [16]. In the *mdx* mouse model, right ventricular dysfunction has also been shown to be an important early feature, and similarly to the clinical studies above, right ventricular dysfunction precedes left ventricular dysfunction [17]. This suggests that the pathophysiology of right ventricular dysfunction is due to an intrinsic right ventricular process as opposed to right ventricular dysfunction developing secondary to left ventricular dysfunction in the setting of secondary pulmonary hypertension and increased right ventricular afterload. The detrimental effect of metroprolol on this early marker of cardiomyopathy in the *mdx* mice would suggest caution for early beta-blocker usage. One potential explanation linking the apparent improvement in left ventricular function and deterioration in right ventricular function is that the relatively low stroke volume from the left ventricle without treatment protects the abnormal and susceptible right ventricle from excessive volume overload. However, the beta-blockers restore stroke volume and cardiac output to wild type levels and this increased flow to the right side of the heart then causes greater dilatation of the right ventricle. This is a phenomenon that is well recognized when patients with biventricular dysfunction have left ventricular output restored with a left ventricular assist device that then aggravates right ventricular dysfunction [18].

### Left ventricular function and mass

Whereas our findings with respect to left ventricular function and beta-blockade in these models are similar to those that we have found with pressure volume loop based conductance catheter studies (especially in the *mdx* mouse), there are clearly differences between the 2 methods [6]. Stroke volume was reduced with the conductance catheter study in the *Sgcd*-/- mice, though is unaffected in the current study relative to C57 Bl10 mice. There are several potential reasons for this. Firstly, a different anaesthetic regimen was previously used (fentanyl, fluanisone and midazolam i.p.) as opposed to isoflurane in the current study, and it is known that different anaesthetic agents can have markedly different effects on cardiac function [19]. Also, the conductance catheter is an invasive procedure which involves some blood loss and an abdominal incision for the inferior vena caval occlusions. Heart rates tended to be higher with the conductance catheter study. In general, as previously recognised, left ventricular volumes are much lower when measured with the conductance catheter [20]–[21]. There are however unique aspects of both of these techniques that make them both useful: MRI allows for assessment of right ventricular function and other aspects of metabolism such as calcium influx with MEMRI; whereas the conductance catheter allows for analysis of pressure-related measurements such as d*P*/d*t*_max and min_ and pressure-volume calculations of left ventricular contractility. Finally, it is possible that the two methods have different sensitivities to detect subtle changes in ventricular volumes. There are potential discrepancies in the *ex-vivo* measurements of heart to body weight ratios and the MRI measurements of left ventricular mass. The heart to body weight ratio involves both right and left ventricles, and with the dilatation of the right ventricle in the *mdx* mouse this is a plausible reason why left ventricular mass is not increased, although heart to body weight ratio is. In the *Sgcd*-/- mice the heart to body weight ratio increases after metoprolol, and this may be reflected in the higher left ventricular volumes relative to untreated *mdx* mice after metoprolol treatment, and is consistent with our previous conductance catheter studies in these mice with metoprolol showing deterioration in systolic and diastolic function.

### *In-vivo* myocardial calcium influx

Increased calcium influx into the cardiac myocyte is an important phenomenon in muscular dystrophy cardiomyopathy leading to progression of left ventricular dysfunction. Recurrent membrane injury leads to an increased influx of calcium [22] which then causes downstream effects such as activation of calcium-dependent hypertrophic pathways [23], reactive oxygen species [24] and cell death through necrosis with mitochondrial defects [25]. MEMRI can be used to non-invasively assess myocardial calcium influx [8], and we have recently shown that it is increased in these 2 mouse models [13]. Despite the similar increase in calcium influx in both models we showed that there were likely different mechanisms responsible for this in the 2 models. In the *Sgcd*-/- mice there was less reduction in contrast enhancement with the L-type calcium channel blocker diltiazem compared to the *mdx* or wild type mice. Also, higher heart rates played some role in the increased contrast enhancement in the *Sgcd*-/- mice, though heart rate was seen not to account for all the increase when this was brought down to wild type levels. The current study suggests that this important pathophysiological mechanism leading to left ventricular dysfunction is unaffected by beta-blockade. We do not know if successful treatment with beta-blockers in situations where it is proven that they have beneficial effects on outcomes (such as adult heart failure) reduces calcium influx, and so the exact relevance of this finding pending further studies is unclear.

### Conclusions

Treatment with the beta-blocker metoprolol at an early stage in the development of the cardiomyopathy leads to worsening right ventricular function in the *mdx* mouse, and in both models has no effect on calcium influx. This suggests that clinical studies with beta-blockers and other heart failure medications should comprehensively evaluate not only left ventricular function but also right ventricular function and other aspect of myocardial metabolism.
